# Single Amino Acid Arginine Deprivation Triggers Prosurvival Autophagic Response in Ovarian Carcinoma SKOV3

**DOI:** 10.1155/2014/505041

**Published:** 2014-06-01

**Authors:** Galyna Shuvayeva, Yaroslav Bobak, Natalia Igumentseva, Rossella Titone, Federica Morani, Oleh Stasyk, Ciro Isidoro

**Affiliations:** ^1^Institute of Cell Biology, National Academy of Sciences of Ukraine, Drahomanov Street 14/16, Lviv 79005, Ukraine; ^2^Laboratory of Molecular Pathology, Department of Health Sciences, Università del Piemonte Orientale, Via P. Solaroli 17, 28100 Novara, Italy

## Abstract

Autophagy is a process of cytosol-to-lysosome vesicle trafficking of cellular constituents for degradation and recycling of their building blocks. Autophagy becomes very important for cell viability under different stress conditions, in particular under amino acid limitation. In this report we demonstrate that single amino acid arginine deprivation triggers profound prosurvival autophagic response in cultured human ovarian cancer SKOV3 cells. In fact, a significant drop in viability of arginine-starved SKOV3 cells was observed when autophagy was inhibited by either coadministration of chloroquine or transcriptional silencing of the essential autophagy protein BECLIN 1. Enzymatic arginine deprivation is a novel anticancer therapy undergoing clinical trials. This therapy is considered nontoxic and selective, as it allows controlling the growth of malignant tumours deficient in arginine biosynthesis. We propose that arginine deprivation-based combinational treatments that include autophagy inhibitors (e.g., chloroquine) may produce a stronger anticancer effect as a second line therapy for a subset of chemoresistant ovarian cancers.

## 1. Introduction

It is established that some types of tumours are deficient in the biosynthesis of certain amino acids and often exhibit elevated sensitivity to deprivation of a corresponding single amino acid (such as arginine, methionine, and asparagine), both* in vitro* and, importantly,* in vivo* (for recent reviews: [1–5]). This provided a rational basis for the development of metabolic anticancer therapies based on the application of recombinant amino acid degrading enzymes, such as asparaginase for the treatment of leukemias and other tumours [2, 5, 6]. First clinical trials with recombinant enzymes hydrolyzing amino acid arginine, human arginase I, and* Mycoplasma hominis* arginine deiminase have demonstrated therapeutic efficacy in controlling the growth of hepatocarcinomas and melanomas [7–9]. Recent* in vitro* studies also suggested that other types of cancers may be potentially sensitive to this therapy (pancreatic, prostate, renal carcinomas, and mesotheliomas) due to the transcriptional silencing of arginine anabolic enzyme of urea cycle, argininosuccinate synthetase (ASS) [10–13] (see Figure 2). It was also observed that the development of chemoresistance to platinum compounds in ovarian carcinomas leads to collateral appearance of arginine auxotrophy due to the downregulation of ASS [14], adding these tumours to the list of potential targets of arginine deprivation-based enzymotherapy.

Although metabolic enzymotherapy based on arginine deprivation is considered as nontoxic and selective, it is not free of certain limitations. One such limitation arises from the upregulation of ASS expression in many tumours in response to arginine starvation, leading to the appearance of the ASS-positive tumour relapse insensitive to the therapy [2]. Also, we recently observed that tumour cells become profoundly more resistant to arginine withdrawal in* in vitro* 3D spheroid models relative to respective monolayer cultures [15, 16]. This phenomenon is consistent with the results of animal studies and ongoing clinical trials which showed that arginine deprivation is effective in inhibiting tumour growth but not in inducing tumour regression. The latter observation stimulates further search for more efficient rational combinational therapeutic approaches based on arginine deprivation.

Arginine, besides being required for protein biosynthesis, has other versatile functions in the cell as a precursor of nitric oxide, agmatine, and polyamines [17]. It was also demonstrated that arginine is an essential amino acid for cultured tumour cells due to their deficiency in arginine biosynthesis* de novo* [18]. Thus, arginine withdrawal profoundly affects tumour cell physiology. In this work we show that arginine deprivation strongly induces the autophagic process in ovarian carcinoma cells in monolayer culture. Autophagy, the selective process of lysosomal recycling of cell constituents, is known to have a prosurvival role under different stresses in tumour cells [19]. Therefore, we addressed the question whether inhibition of autophagy affects tumour cell survival upon arginine starvation. Such a strategy could be applied to enhance the therapeutic effect of enzymotherapy based on arginine deprivation.

## 2. Materials and Methods

### 2.1. Reagents

The following antibodies were used: polyclonal antibodies against MAP-LC3 (Novus Biologicals, CO, USA) and BECLIN 1 (BD Biosciences, CA, USA), mouse monoclonal anti-LAMP1 (BD) and anti-Golgin97 (Santa Cruz Biotechnology, Santa Cruz, CA, USA), monoclonal mouse anti-*β*-actin (Sigma-Aldrich, St. Louis, MO, USA), ph-4E-BP1 and ph-p70-S6k (Cell Signaling Technology, Beverly, MA, USA), FITC-conjugated polyclonal goat anti-rabbit (Santa Cruz) and Cy3-conjugated polyclonal goat anti-mouse (Santa Cruz), and horseradish peroxidise- (HRP-) conjugated polyclonal goat anti-mouse and anti-rabbit (both from Millipore Corporation, Bedford, MA, USA).

Monodansylcadaverine (MDC), 3-methyladenine (3MA), chloroquine (CQ), asparagine (Asn), and other bench chemicals were purchased from Sigma-Aldrich.

### 2.2. Cell Line and Culture Conditions

SKOV3 cells originating from human ovarian carcinoma tissues were obtained from ATCC (USA). The cells were grown in Dulbecco's modified Eagle's medium (DMEM; HyClone Laboratories, Logan, Utah, USA) with 10% foetal bovine serum (FBS; PAA Laboratories GmbH, Pasching, Austria), 2 mmol/L glutamine, and 50 mg/L gentamycin (Sigma-Aldrich, Steinheim, Germany) and maintained in the incubator at 37°C with 5% CO_2_. Where indicated, arginine-containing (0.4 mM; HyClone Laboratories, Logan, UT, USA) and arginine-free media were supplemented with 5% dialysed FBS (HyClone). To study the growth dynamics of ovarian carcinoma cells under standard and arginine-deprived conditions, the cells were seeded at a density of 20000 cells per well in regular medium in 96-well plates and allowed to adhere for 24 h; then the medium was aspirated and the cell monolayer was washed two times with PBS, and finally the cells were incubated with fresh complete medium or arginine-free medium (AFM). The cells were cultured for up to 96 h, and cell growth was assessed by counting the cells every 24 h in triple. Cell viability was assessed using the trypan blue (final concentration 0.05%) dye exclusion. Viable (unstained) and nonviable (blue-stained) cells were counted on a haemocytometer by light microscopy.

### 2.3. RT-PCR

Total RNA was isolated from cells by the method of Chomczynski and Sacchi [20]. First-strand cDNA synthesis was performed using First Strand cDNA Synthesis Kit (Fermentas, Vilnius, Lithuania) and an oligo-dT primer according to the manufacturer's instructions. PCR was performed using a High Fidelity PCR Enzyme Mix (Fermentas) with the following primer pairs:

 ASS-S, 5′-GGGGTCCCTGTGAAGGTGACC-3′;
 ASS-AS, 5′-CGTTCATGCTCACCAGCTC-3′;
 ASL-S, 5′-GAAGCGGATCAATGTCCTGC-3′;
 ASL-AS, 5′-CTCTTGGTGAATCTGCAGCG-3′;
 OTC-S, 5′-AATCTGAGGATCCTGTTAAACAATG-3′;
 OTC-AS, 5′-CTTTTCCCCATAAACCAACTCA-3′;
 GAPDH-S, 5′-CAAGGTCATCCATGACAACTTTG-3′;
 GAPDH-AS, 5′-GTCCACCACCCTGTTGCTGTAG-3′.

PCR fragments were separated by electrophoresis on 1.5% agarose gel and visualized by ethidium bromide staining. The relative mRNA expression levels were estimated after normalization with GAPDH. The number of cycles was chosen at which PCR product amount was optimal and within the linear portion of the curve, well before saturation point.

 ASS: number of cycles—26, size of the amplicon—448 bp,
 ASL: number of cycles—27, size of the amplicon—502 bp,
 OTC: number of cycles—40, size of the amplicon—1125 bp,
 GAPGH: number of cycles—25, size of the amplicon—496 bp.

### 2.4. Visualization of Monodansylcadaverine-Labelled Vacuoles

MDC is an autofluorescent weak base that accumulates in acidic lysosomal vacuoles and autophagolysosomes [21]. Cells attached to glass coverslips were incubated with 0.05 mM MDC (Sigma-Aldrich) in PBS at 37°C for 10 min. After incubation, cells were washed three times with PBS and immediately analyzed with a fluorescence microscope (ZEISS, Axio Imager A1) equipped with Axio Vision Software (v. 4.6.3). Images were captured with a CCD camera and imported into Photoshop. Quantification of cell fluorescence was conducted using ImageJ 1.48v Software.

### 2.5. Immunofluorescence and Microscopy Analysis

Immunofluorescence staining was performed as previously described [22]. Essentially, cells cultured on glass coverslips were washed with PBS, fixed with cold methanol, and permeabilized with 0.2% Triton X-100 in PBS. The coverslips were then incubated with the indicated primary antibodies in PBS contained 0.1% Triton X-100 and 4% FBS overnight at 4°C and thereafter incubated with the appropriate secondary antibodies for 1 hr at room temperature. Nuclei were stained with DAPI. Coverslips were mounted on microscope slides and monitored under a ZEISS fluorescence microscope (Axio Imager A1) equipped with Axio Vision Software (v. 4.6.3). Images were captured with a CCD camera and imported into Photoshop. Pearson correlation coefficients were calculated using ImageJ 1.48v Software to assess the degree of colocalization of protein markers of autophagy.

### 2.6. Immunoblotting

The cell monolayer was washed with ice-cold PBS and lysed in Extraction Buffer (10 mmol/L Tris-HCl, pH 7.5, 150 mmol/L NaCl, 1% NP-40, 5 mmol/L EDTA, 50 mmol/L NaF, 1 mmol/L Na_3_VO_4_, 5 mmol/L benzamidine, 1 mmol/L PMSF, 10 mg/mL aprotinin, 10 mg/mL Leupeptin, and 1 mg/mL Pepstatin A) at 4°C for 20 min. Cell extracts were obtained after centrifugation at 12,000 g at 4°C for 30 min and cellular proteins were quantified using Peterson's method [23]. Equal amounts of protein homogenates were loaded, separated by SDS-PAGE (concentration of acrylamide varied depending on the size of the protein to be detected), and transferred onto PVDF membrane (Millipore Corp., Billerica, MA, USA). The membranes were blocked with 5% nonfat dried milk in PBS containing 0.05% Tween-20 and probed with the indicated primary and secondary (horseradish peroxidase-conjugated) antibodies. *β*-Actin was used for protein loading control. The bands were visualized using the enhanced chemiluminescence reagent (Millipore Corp.). Band densitometry quantification was performed using the Gel-Pro analyzer (Version 32).

### 2.7. Small Interfering RNA Transfection

BECLIN 1 silencing was achieved using RNA interference as previously described [24]. Cells were transfected using Oligofectamine reagent (Invitrogen, Carlsbad, CA, USA) according to the manufacturer's manual. Protein knockdown was determined by immunoblotting 48 hr after transfection. As control (sham transfection), a nonspecific scramble sequence siRNA was used.

### 2.8. Statistics

In each individual experiment triplicate wells were used for each treatment and control. All experiments were repeated at least three times. Statistical analyses were performed using Student's* t*-test. Results were expressed as means ± SD. Significance was established when the* P* value was less than 0.05.

## 3. Results

### 3.1. SKOV3 Cells Retain Viability and Proliferative Potential under Long-Term Arginine Deprivation* In Vitro*

**Figure 1:**
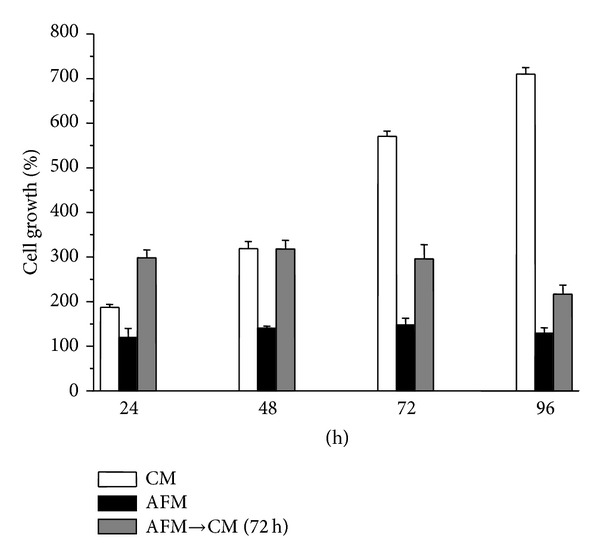
Effect of arginine deprivation on SKOV3 cell proliferation. The growth of SKOV3 cells in arginine-sufficient complete medium (CM), in arginine-free medium (AFM), and the ability of SKOV3 cells to rescue cell proliferation after different periods of arginine starvation was assessed by counting only the viable cells. After the indicated periods of incubation in AFM, cells were shifted to a fresh CM and allowed to proliferate for additional 72 h (AFM→CM (72 h)). Viable cells were determined by the trypan blue exclusion test. The initial number of cells (time-point zero) was considered as 100%. Data from three independent experiments in triplicate.

Tumour cell lines in monolayer cell culture substantially differ in their sensitivity to a single amino acid withdrawal [18]. Therefore, we first analyzed whether and to what extent arginine deprivation affects the viability and the proliferative potential of SKOV3 cells. Upon shifting to a defined arginine-deficient medium (AFM), growth arrest and reduction in the proportion of viable cells were observed. It is to be noted that, even after 4 days of arginine starvation, SKOV3 cells were still able to resume cell proliferation in response to arginine resupplementation, though regrowth potential progressively declined in the course of incubation in AFM (Figure 1). No signs of PARP fragmentation as a reporter of apoptosis in arginine-starved SKOV3 cells were observed (data not shown). This observation suggested that a substantial fraction of SKOV3 cells remained viable even after the prolonged arginine withdrawal indicating that these cells are rather resistant to this metabolic stress. For comparison, hepatocellular carcinoma HepG2 cells lose their proliferative potential already after 2 days of arginine starvation and exhibit concomitant apoptosis [18].

### 3.2. Arginine Is an Essential Amino Acid for Cultured SKOV3 Cells

**Figure 2:**
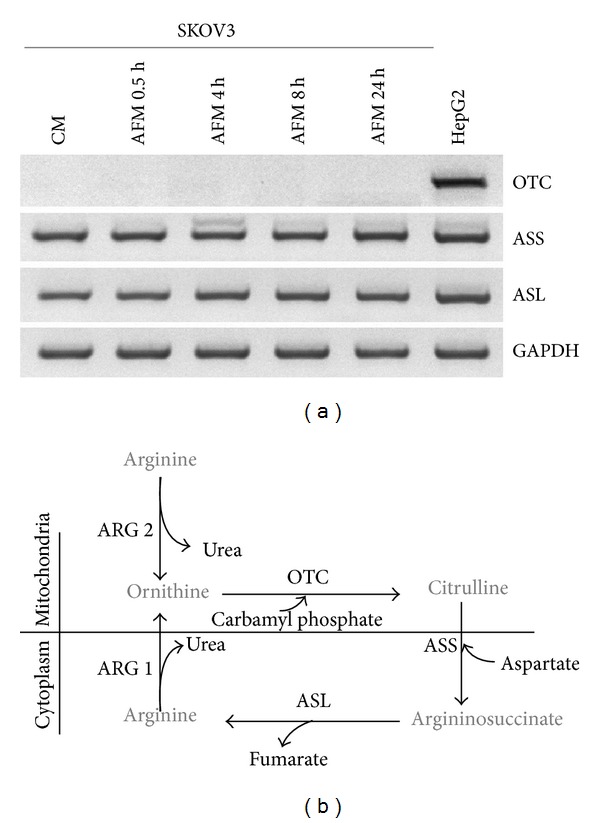
Expression of the key genes of arginine anabolism in SKOV3 cells. (a) Specific mRNA levels determined by RT-PCR analysis as described in Section 2. Cells were cultured in arginine-free medium (AFM) for 24 h or in arginine-sufficient complete medium (CM). Human hepatocarcinoma HepG2 cells (which express urea cycle enzymes) were used as a positive control. GAPDH expression was used as an internal loading control. (b) Scheme of arginine biosynthesis in the urea cycle. ARG1: arginase I, ARG2: arginase II, OTC: ornithine transcarbamylase, ASS: argininosuccinate synthetase, and ASL: argininosuccinate lyase.

The effect on growth arrest in AFM (Figure 1) suggested that arginine is an essential amino acid for SKOV3 cells. The RT-PCR analysis of arginine key anabolic enzymes of the urea cycle revealed that SKOV3 cells incubated in complete medium do not express mitochondrial arginine biosynthetic enzyme ornithine transcarbamylase (OTC; it converts ornithine to citrulline) but do express cytosolic argininosuccinate synthetase (ASS; it converts citrulline to argininosuccinate) and argininosuccinate lyase (ASL; it converts argininosuccinate to arginine) (Figures 2(a) and 2(b)). Arginine deprivation did not trigger an upregulation of ASS or ASL, often observed in other tumour cell lines, or induction of OTC transcription (Figure 2(a)). Human hepatocellular carcinoma HepG2 cells were used as a positive control [18]. OTC deficiency in cultured SKOV3 cells implies that they are deficient in endogenous arginine anabolism, and arginine can only be synthesized via ASS-mediated conversion of exogenously supplied citrulline (which is absent in standard DMEM medium). Accordingly, exogenous ornithine did not support proliferation of SKOV3 cells in AFM (data not shown). Therefore, we can assume that incubation in AFM surely induces arginine starvation in SKOV3 cells.

### 3.3. Arginine Deprivation in SKOV3 Cells Triggers Profound Autophagic Response

**Figure 3:**
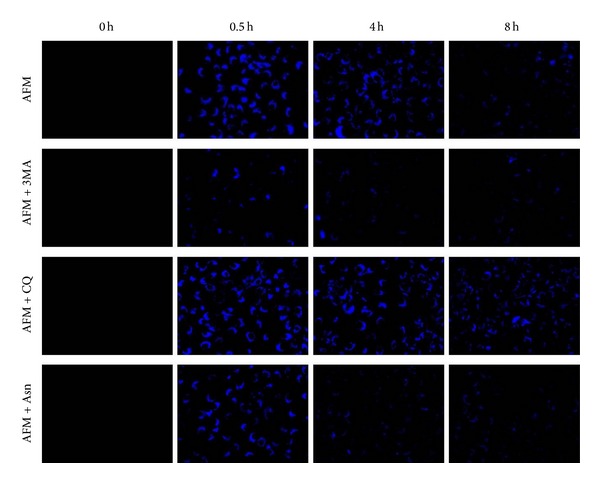
MDC staining of acidic vacuoles in ovarian carcinoma SKOV3 cells subjected to arginine deprivation (AFM). The cells were labelled with MDC as described in Section 2 at zero point and after 0.5, 4, and 8 hours of arginine withdrawal and immediately monitored under a fluorescence microscope. Magnification 400x.

One of the prosurvival responses of tumour cells triggered upon amino acids limitation is the elevated intracellular protein recycling via autophagy [19]. In particular, arginine deprivation has been shown to induce autophagy in melanomas [25]. However, it is known that different tumour cells exhibit varying basal and stimulus-dependent inducible autophagic proficiency [26]. We addressed the question whether low sensitivity (in terms of cell survival and proliferation) of SKOV3 cells to arginine withdrawal as an essential amino acid (Figure 1) was associated with, or causally linked to, induction of autophagy. To monitor autophagy, we first employed a classical staining of acidic vacuoles, which includes lysosomes and autophagolysosomes, with the vital fluorescent dye MDC [21]. Arginine deprivation rapidly and profoundly induced the expansion of the autophagolysosomal compartment in SKOV3 cells (Figure 3). We observed MDC-labelled intracellular vacuoles in SKOV3 cells already after 30 min of arginine starvation. Treatment of the starved cells with 3-methyladenine (3MA, 10 mM), a classic inhibitor of autophagy that inhibits the formation of autophagosomes [22], strongly diminished the fluorescence signal (Figure 3). Upon treatment with chloroquine (CQ, 25 *μ*M), another known inhibitor of autophagy [21], MDC staining of the cells further increased (Figure 3). This effect was expected, as CQ impairs the late stages of autophagy and leads to the accumulation of autophagosomes and of autophagolysosomes [21]. By contrast, a partial decrease in MDC staining in arginine-deprived SKOV3 cells was observed upon concomitant treatment with an excess of asparagine (Asn, 50 mM), which is known to downregulate autophagy through the stimulation of mTOR, a negative regulator of autophagy [21]. Quantification of cell fluorescence under arginine starvation and upon cotreatment with inhibitors using ImageJ 1.48v Software revealed an approximately four-time decrease in fluorescence between control arginine-starved and CQ-treated cells versus those treated with 3MA and Asn at 4 hours of incubation (data not shown). Taken together, the above data indicate that MDC staining is indeed mirroring the induction of autophagolysosomes in arginine-deprived SKOV3 cells. To definitively demonstrate the induction of autophagy, in a parallel experiment the cells cultured on coverslips were immunostained for LC3 (a hallmark of autophagosomes) and LAMP1 (a marker of lysosomes), as well as for BECLIN 1 (a component of the autophagy interactome) and Golgin97 (which labels the Golgi complex). Colocalization of LC3 and LAMP1 is a reliable indicator for the formation of autophagolysosomes, while formation of BECLIN 1 aggregates in the Golgi area is indicative of activation of the autophagy process [24].

**Figure 4:**
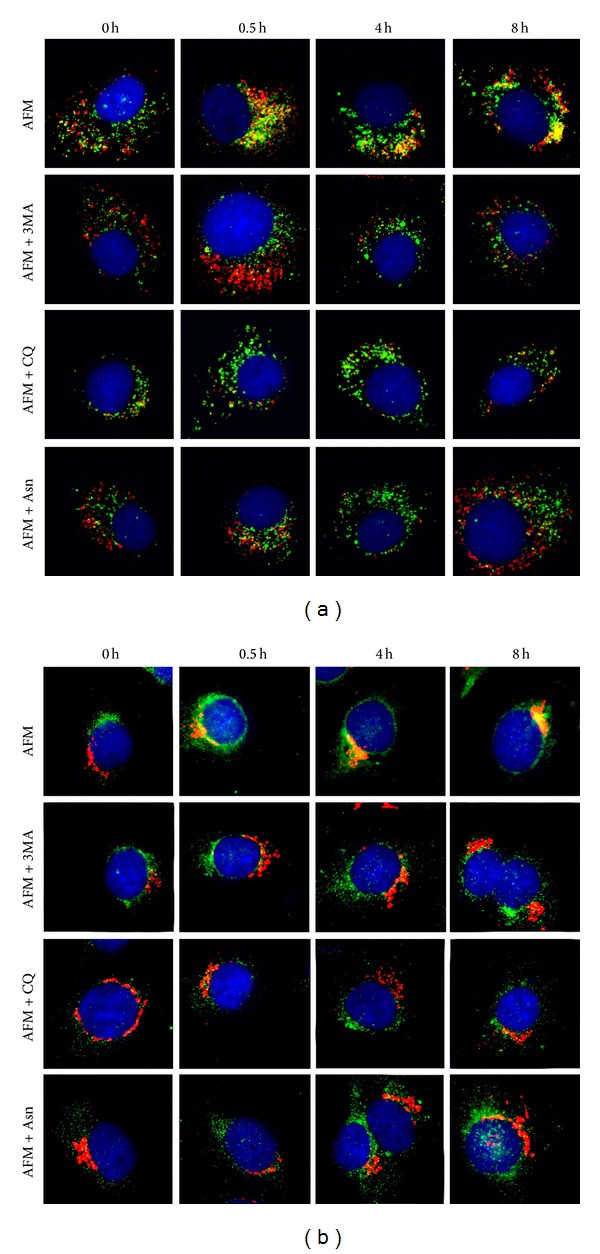
(a) Immunofluorescence staining of the autophagosomal protein LC3 (green fluorescence) and of the lysosomal protein LAMP1 (red fluorescence) and (b) of BECLIN 1 (green fluorescence) and Golgi-associated Golgin97 (red fluorescence) in SKOV3 cells subjected to arginine starvation. Nuclei were labelled with DAPI. Images were captured with ZEISS fluorescence microscope (Axio Imager A1) equipped with Axio Vision Software v. 4.6.3. Magnification 1000x.

We observed the colocalization of such signals (yellow fluorescence) already at 30 min of arginine deprivation (Figures 4(a) and 4(b)), indicating a fast upregulation of the autophagy process. Colocalization of autophagosome- and lysosome-associated proteins was evident during the whole time course of our analysis (Figures 4(a) and 4(b)). Importantly, in agreement with the MDC data (Figure 3), 3MA and Asn reduced while CQ increased the number and the size of LC3-positive vacuoles (i.e., autophagosomes) and also the aggregation of BECLIN 1 in the Golgi area (Figures 4(a) and 4(b)). The calculated values of Pearson correlation coefficient for the pairs LC3/LAMP1 and BECLIN 1/Golgin97 were ≥0.5 for arginine-starved cells, whereas for cells treated with inhibitors (3MA, CQ, and Asn) the coefficients values were 0 to ≤0.2. From these data we conclude that arginine deprivation strongly induces an autophagic response in ovarian carcinoma SKOV3 cells.

### 3.4. Effects of Arginine Deprivation and Autophagy Impairment on mTOR-Dependent Biosynthesis Pathway

**Figure 5:**
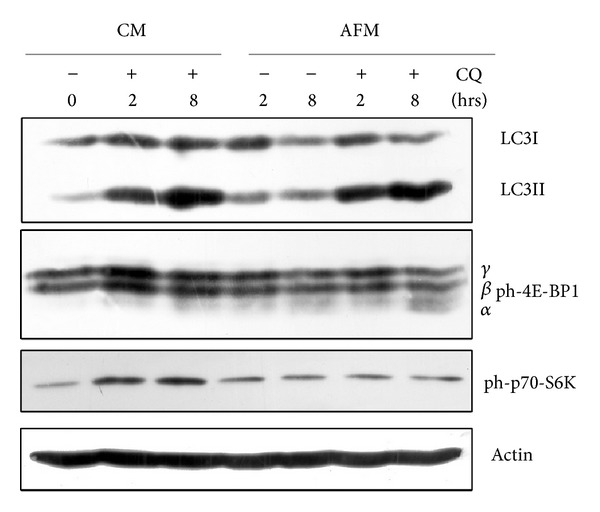
Effect of arginine deprivation and CQ treatment on accumulation of autophagosomal protein LC3II and phosphorylation of mTOR substrates. After indicated periods of treatment, the cells were washed and harvested for WB analysis. 50 *μ*g of total sample protein was loaded per lane. *β*-Actin served as the loading standard.

Amino acid starvation is known to inhibit the biosynthetic pathway and, in parallel, to induce the autophagy degradation of redundant protein as an attempt to rescue the amino acids needed for the synthesis of vital proteins. The mTOR kinase is placed at the cross-point and is a master regulator of both these pathways [27, 28]. To get an insight into the relationship between the induction of autophagy and the biosynthetic pathway under arginine-deprivation conditions, we assayed the activation status of 4E-BP1 (eukaryotic initiation factor 4E-binding protein 1) and of p70-S6K (ribosomal p70 S6 kinase), two downstream substrates of mTOR that direct protein synthesis [29], in the presence of CQ, that hampers the last step of autophagy. In fact, in the presence of CQ the consumption of autophagosomes is interrupted as witnessed by the induced accumulation of LC3II both in CM and in AFM conditions, and this accumulation increases with time of incubation (Figure 5). However, in the cells subjected to arginine deprivation the level of LC3I decreases with time of incubation despite the fact that no further increase in LC3II is observed, indicating a rapid autophagy flux and consumption of autophagosomes. When these cells were concomitantly exposed to CQ the autophagosomes in fact accumulated with time (as indicated by increased LC3II at 8 versus 2 h). Next we looked at the translational activity in SKOV3 cells cultivated under these conditions. While phosphorylation of 4E-BP1 elicits its inhibition and therefore reliefs the inhibitory action of 4E-BP1 on protein synthesis, the phosphorylation of p70-S6K elicits the activation of the translational process [29]. Thus, the phosphorylation of both 4E-BP1 and p70S6k converges on triggering the initiation of protein synthesis. Both 4EBP and p70-S6K were highly phosphorylated in the cells cultivated in CM in the presence of CQ, while their activation status was greatly reduced under AFM culture condition regardless of whether CQ was or was not present (Figure 5).

### 3.5. Effect of Autophagy Inhibition by Transcriptional Silencing of BECLIN 1 or with CQ on the Sensitivity of SKOV3 Cells to Arginine Starvation

**Figure 6:**
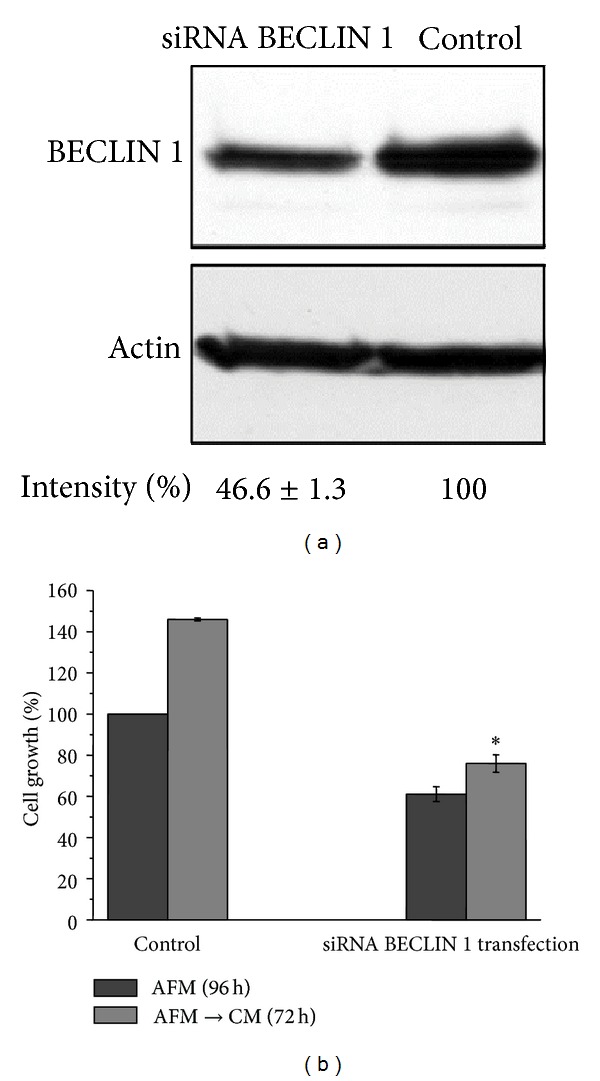
Transcriptional silencing of BECLIN 1 expression leads to decreased viability of SKOV3 cells under arginine starvation. (a) Western blot analysis of BECLIN 1 protein in SKOV3 cells transfected with BECLIN 1 siRNA or with nonsilencing control siRNA. *β*-Actin served as the loading standard. Densitometry quantification and the calculation of the relative band intensity were performed as described in Section 2. Intensity represents the mean value of three independent experiments and is expressed as means ± SD; (b) histogram represents the effect of BECLIN 1 silencing on the viability of arginine-starved cells and their proliferative potential after arginine resupplementation. *P < 0.05.

To elucidate the role of autophagy in maintaining SKOV3 cell viability under arginine deprivation, we manipulated the autophagy pathway by genetic and pharmacologic approaches. Transient transfection with a specific siRNA elicited the transcriptional silencing (approximately 54%) of the autophagy protein BECLIN 1 (Figure 6(a)). After three days of incubation in AFM, the number of viable cells in siRNA-BECLIN 1 transfected culture decreased by approximately 40% as compared to the sham-transfected culture (Figure 6(b)). Arginine-deprived BECLIN 1-silenced cells also exhibited a decrease in their proliferative potential upon shift to the fresh CM relative to control cells (Figure 6(b)), thus suggesting a significant prosurvival role of autophagy in the cell response to arginine starvation.

**Figure 7:**
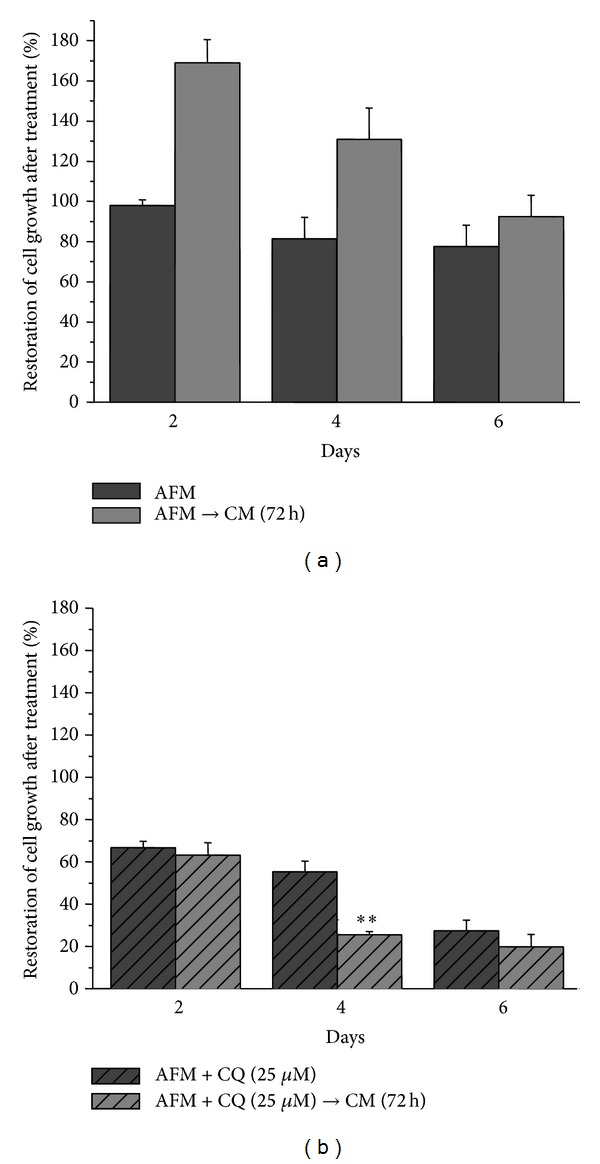
Effect of the autophagy inhibitor chloroquine (CQ) on SKOV3 cells viability and proliferative potential. Histograms showing cell survival and ability to resume proliferation after arginine resupplementation in cells deprived of arginine (AFM) (a) or cotreated with CQ (b). After the indicated periods of the treatment, the medium was changed to CM and cells were allowed to grow for additional 72 h. Viable cell numbers were determined by the trypan blue exclusion test. 100% is the number of cells at zero time point. **P < 0.01.

Next, we addressed the question whether cotreatment with CQ, which impairs the late steps of the autophagy pathway, affects the survival of SKOV3 cells under arginine deprivation in a similar manner as observed with the silencing of BECLIN 1 expression. To this end, we monitored cell viability under the combined treatment and cell proliferation upon arginine resupplementation. The treatment with CQ (25 *μ*M) dramatically decreased cell viability and the proliferative potential under arginine starvation (Figure 7). Importantly, CQ treatment rendered arginine-starved SKOV3 cells unable to resume proliferation in fresh CM already after two days of the combined treatment (Figure 7; cf. Figure 1). It is to be noted that a prolonged incubation with this same concentration of CQ was cytotoxic for SKOV3 cells also in CM medium (data not shown). No signs of apoptosis (as tested by western blotting of PARP and Annexin V staining) and no signs of senescence (as tested by positivity for beta-galactosidase staining) were detected in AFM plus CQ cotreated cells up to 144 h. However, SKOV3 cells double staining with ethidium bromide and Hoechst 33342 revealed that under this culture condition the majority of cells died via necrosis (not shown).

## 4. Discussion

Autophagy, a cytosol-to-lysosome membrane-trafficking process of degradation of cellular constituents, is a housekeeping homeostatic pathway and also plays a fundamental role to preserve cell viability upon different stress conditions [19]. One of such stresses is nutrient limitations, in particular amino acid restriction [30]. Autophagy is known to have dual function in cancerogenesis, playing both negative and positive roles in cancer progression and being implicated in chemoresistance of certain tumour types (for review: [26, 31–33]). It was also established that even single amino acid starvation triggers autophagic response in tumour cells [11, 34]. Previously reported [35] and our unpublished data suggest that the most profound autophagic response in tumour cells is triggered by starvation for arginine, methionine, lysine, and leucine relative to other amino acids. It remains to be elucidated whether specific regulation by this set of amino acids involves the mTORC1 complex or other mechanisms. Our data indicate that single arginine deprivation early affects the mTOR-dependent biosynthetic pathway.

Arginine, besides being required for protein biosynthesis, has other versatile functions in the cell as a precursor of nitric oxide, agmatine, and polyamines and as a regulatory molecule (for review: [17]). For cultured tumour cells arginine is an essential amino acid due to their deficiency in arginine biosynthesis* de novo* [18]. Ovarian carcinomas were recently added to the growing list of tumour types potentially sensitive to the treatment with recombinant arginine-degrading enzymes. In fact, it was demonstrated that relapses of ovarian carcinomas resistant to cisplatinum treatment concomitantly become deficient in argininosuccinate synthetase, a rate limiting enzyme of arginine biosynthesis, and thus potentially sensitive to arginine-degrading enzymes [14]. In this work we investigated how modulation of autophagy affects ovarian cancer cells viability under arginine deprivation. Human ovarian carcinoma SKOV3 cells were used as an experimental model. We found that SKOV3 cells exhibit high resistance to the stress exerted by arginine deprivation (Figure 1). This fact potentially allows studying the physiological role of autophagy under arginine withdrawal without interfering with processes of programmed cell death (apoptosis) that are often triggered to a different extent in cancer cells under such conditions [18]. Although SKOV3 cells exhibit high expression of argininosuccinate synthetase (as we show in Figure 2), they still are a suitable informative model for* in vitro* studies since SKOV3 cells are fully dependent on exogenous arginine supply due to the deficiency in ornithine transcarbamylase (OTC), an upstream enzyme of arginine anabolism (Figure 2). In this report we demonstrate that arginine withdrawal rapidly and markedly induces autophagy in SKOV3 cells (Figures 3 and 4). Under starvation, the biosynthetic pathway is impaired while basal autophagy rises up, and both these pathways are controlled by mTOR [27, 36]. Autophagy fuels the cytoplasm with the amino acids deriving from proteolysis and CQ is expected to interrupt this process by impairing the formation of autophagolysosomes and by inhibiting the acid-dependent proteolysis mediated by lysosomal cathepsins. The mTORC1 complex senses the availability of amino acids and phosphorylates downstream substrates in order to switch on and off the pathways for autophagy degradation or for protein synthesis accordingly [28]. Arginine deprivation in fact depressed the activation of the signalling kinases 4E-BP1 and p70-S6k that govern the protein synthesis pathway (Figure 5). These kinases are downstream of mTOR [29], which also negatively controls autophagy. CQ, which further reduces the availability of autophagy-derived amino acids, affected the signalling that governs the biosynthetic pathway in the cells cultivated in CM, indicating that despite the presence of amino acids in the culture medium the block of the autophagy degradation imposed by chloroquine was sensed by mTOR. By contrast, in AFM the mTOR pathway was inactive since the first 2 h of incubation and CQ did not reduce further the level of phosphorylation of 4E-BP1 and p70-S6k, indicating that arginine deprivation* per se *was sufficient to limit or inhibit the activation of the protein synthesis pathway. We also demonstrate that autophagy process is important for maintaining cell viability under arginine deprivation. This conclusion is supported by the significant drop in viability of arginine-starved SKOV3 cells in which autophagy is inhibited. In this respect, either coadministration of CQ or transcriptional silencing of the essential autophagy protein BECLIN 1 produced similar effects (Figures 6 and 7). Strikingly, in the case of BECLIN 1 siRNA silencing, the observed decrease in viability and proliferative potential was roughly proportional to the remaining level of BECLIN 1 protein in the transfected culture (Figure 6). Preliminary data from our laboratories indicate that coapplication of taxane (taxol) at low doses may further decrease viability of ovarian carcinoma SKOV3 cells under arginine deprivation (to be published elsewhere). In this context, it is to be noted that taxol is a disruptor of the cytoskeleton and negatively impacts on the autophagosome-lysosome fusion step. In conclusion, our data support the conception that combinational treatment based on arginine deprivation and an autophagy inhibitor (e.g., chloroquine, a known nontoxic antimalarial drug) can potentially be applied as a second line treatment for a subset of ovarian carcinomas deficient in ASS.
